# Synchronous Hepatic Cryotherapy and Resection of Colonic Primary is a High Risk Procedure

**DOI:** 10.1155/2000/60432

**Published:** 2000-08

**Authors:** D. Cheung, D. L. Morris

**Affiliations:** The University of New South Wales Department of Surgery St. George Hospital Sydney Kogarah NSW 2217 Australia

## Abstract

Thirteen patients underwent hepatic cryotherapy and synchronous colonic resection. Two of the nine patients developed hepatic abscess – this is a rare complication of cryotherapy alone.


*HPB Surgery*, 2000, Vol. 11, pp. 379–382
Reprints available directly from the publisher
Photocopying permitted by license only

© 2000 OPA (Overseas Publishers Association) N.V.
Published by license under
the Harwood Academic Publishers imprint,
part of The Gordon and Breach Publishing Group.
Printed in Malaysia.

### Synchronous Hepatic Cryotherapy and Resection of Colonic Primary is a High Risk Procedure

D. CHEUNG and D. L. MORRIS*

*The University of New South Wales, Department of Surgery, St. George Hospital, Kogarah NSW 2217, Sydney, Australia*

*(Received 19 May 1999)*

**Thirteen patients underwent hepatic cryotherapy and synchronous colonic resection. Two of the nine patients developed hepatic abscess – this is a rare complication of cryotherapy alone.**

*Keywords:* Liver metastases, colorectal cancer, cryotherapy

### INTRODUCTION

Liver metastases are present in 15–25% of patients at the time of diagnosis [1]. In patients with liver only disease treatment by synchronous hepatic artery catheterization and liver resection are already well described [2–4]. Cryotherapy is the best established method of imaging controlled destruction for unresectable liver tumours [5]. The aim of this study was to review our small experience of synchronous hepatic cryotherapy and colectomy.

### METHOD

Thirteen patients who had synchronous hepatic cryotherapy and colectomy were identified from our database of 1,385 patients with liver cancer including 799 with liver metastases from colorectal cancer treated between 1990 and July, 1998. Patients were treated with cryotherapy if the liver metastases were not resectable but there were a small number of lesions (4 or less) and no extrahepatic disease. Eight of the nine patients who underwent hepatic cryotherapy also had intrahepatic chemotherapy by hepatic artery catheterization. In three of our patients the cryotherapy was applied to a liver resection margin at the time of colectomy. Retrospective reviews of the case notes of the patients were done concentrating on the operative procedure and postoperative recovery.

### TECHNIQUE

After colectomy was completed, the peritoneal cavity was irrigated with two litres of warm normal saline solution mixed with 160 mg of Gentamicin. The patient was then reprepped and draped and a new set of clean instruments used for the hepatic part of the operation. We

*Corresponding author. Tel.: +61 2 9350 2070, Fax: +61 2 9350 3997, e-mail: David.Morris@unsw.edu.au use a standard 5 mm spike cryoprobe directly to the lesion or resection margin using intraoperative ultrasound control [12]. For small intraparenchymal lesions, a spinal needle is placed in the centre of the tumour using ultrasound guidance, and the angle and depth checked. The cryoprobe is then inserted into the liver using ultrasound guidance [12]. Liquid nitrogen circulates through the probe and the tumour is frozen with a margin of at least 1 cm of the surrounding liver tissue in all three dimensions. After an adequate amount of tissue has been frozen, passive thaw of the iceball is allowed by stopping the flow of the liquid nitrogen and leaving the cryoprobe *in situ* to allow a double freeze-thaw cycle. Incomplete double freeze-thaw cycles were used for all patients with a 1 cm edge thaw except one patient who received only single freeze-thaw, because of relatively bulky disease (6 hepatic lesions) with the maximum diameter of the lesions being 6 cm. After the cryoprobe has been removed, gelfoam was placed in the probe tract to prevent bleeding.

### RESULTS

A total of 13 patients had hepatic cryotherapy at the same time as colectomy. Nine patients had hepatic cryotherapy as the principal treatment of their liver metastases, whereas three patients had hepatic cryotherapy to the margins of a liver resection (edge cryotherapy). Eight patients out of the nine patients who underwent hepatic cryotherapy also had a catheter placed for regional chemotherapy. The types of colorectal resection are detailed in Table I and involved left sided or rectal resection in seven patients. The number of hepatic lesions is also detailed in Table I, together with the follow up of our patients. It is difficult to derive any knowledge of the long term results of synchronous cryotherapy and colectomy from our small report. We do have six patients alive and two are tumor free.

The most important finding of this study was that two of nine patients developed postoperative hepatic abscess which is a rare complication following hepatic cryotherapy alone. The first patient was a 78 year old female who underwent right hemicolectomy, hepatic cryotherapy and insertion of hepatic arterial catheter for a caecal tumour with concurrent liver metastases. She had hepatic cryotherapy to six liver metastases. This patient developed a subhepatic abscess that required operative drainage and removal of the hepatic artery catheter three weeks after the initial operation. The patient suffered a protracted course of septic complications with recurrent abscesses and succumbed ten weeks later after active treatment was withdrawn. The second patient, a 53 year old man, underwent an anterior resection, hepatic cryotherapy and insertion of hepatic artery catheter for a proximal rectal tumour. He had three liver metastases that were treated with cryotherapy. He developed a colonic anastomotic leakage eight days after the initial operation causing faecal peritonitis requiring the formation of an end colostomy. He developed liver abscesses at three weeks requiring another laparotomy for debridement of these lesions and removal of the hepatic artery catheter. He was hospitalized for a total of 53 days but recovered and has no evidence of hepatic disease.

### DISCUSSION

Patients with nonresectable liver metastases from colorectal cancer may benefit from hepatic cryotherapy as a local ablative technique [6–10]. The results of hepatic cryotherapy in almost 1,000 patients have been published [11]. Cryotherapy was used in seven out of nine patients because of bilobar liver metastases. The other two patients had cryotherapy as the lesions were in close proximity to major intrahepatic vessels. The three patients who had edge-cryotherapy had inadequate resection margins following liver resection.

TABLE I

| | Name | Age | Do OT | Colectomy | HAC | L'Resect | Cryo | No. | Max dm Cm | Survival | Complication |
|---|---|---|---|---|---|---|---|---|---|---|---|
| 1 | SP | 65 | 1/11/90 | R hemi | Yes | No | Lesion | 7 | 4 | DOD 3/93 | |
| 2 | KL | 53 | 18/7/91 | Sigmoid | Yes | No | Lesion | 4 | 5 | DOD 13/8/93 | |
| 3 | SS | 78 | 24/7/92 | R hemi | Yes | No | Lesion | 6 | 6 | DOD 10/92 | Subhep abscess |
| 4 | RM | 56 | 17/8/93 | R hemi | Yes | No | Lesion | 6 | 5 | DOD 7/95 | |
| 5 | MH | 56 | 24/5/95 | AP | Yes | Yes | Lesion | 1 | 2 | DOD 11/96 | |
| 6 | DN | 51 | 8/4/96 | R hemi | No | No | Lesion | 1 | 5 | DOD 3/8/97 | |
| 7 | JB | 70 | 4/9/96 | AP | Yes | No | Lesion | 5 | 3 | AWD | |
| 8 | BH | 53 | 9/9/97 | Ant resect | Yes | No | Lesion | 3 | 3 | AWD | Hep abs/anast leak |
| 9 | AZ | 78 | 17/3/98 | Ant rec | Yes | Yes | Lesion | 1 | 2 | AND | Hypotension, ileus |
| 10 | ET | 58 | 14/2/98 | Ant resect | No | Yes | Edge | | | AND | |
| 11 | PW | 42 | 3/5/95 | Ant resect | No | Yes | Edge | | | AWD | Bile collection, sump fell out |
| 12 | JP | 48 | 20/9/93 | L hemi | No | Yes | Edge | | | AWD | CVC infection |

AND – Alive do disease.
AWD – Alive with disease.
DOD – Dead of disease.

Cryotherapy is safe, the post-operative mortality has been estimated to be in the proximity of 1.6% [11]. Hepatic abscess is a rare event following cryotherapy occurring in less than 2% of patients [11]. We think that leaving a devitalized frozen liver tissue at the time of colectomy is, however, probably dangerous, as hepatic abscess occurred in two out of nine patients in our series. However, in one patient, the hepatic abscess was probably related to the development of an anastomotic leak and faecal peritonitis. Our small experience of concurrent colectomy and hepatic cryotherapy suggest a relatively high risk of hepatic abscess formation.

### *References*

[1] Ballantyne, G. H. and Qin, J. (1993). Surgical treatment of liver metastases in patients with colorectal cancer. *Cancer* (Suppl.) 71, 4252–66.

[2] Hughes, K., Scheele, J. and Sugarbaker, P. H. (1989). Surgery for colorectal cancer metastatic to the liver. Optimizing the results of treatment. *Surg. Clin. North Am.*, **69**, 339–59.

[3] Bradpiece, H. A., Benamin, I. S., Halevy, A. and Blumgart, L. H. (1987). Major hepatic resection for colorectal liver metastases. *Br. J. Surg.*, **74**, 3224–6.

[4] McCall, J. L., Jorgensen, J. O. and Morris, D. L. (1995). Hepatic artery chemotherapy for colorectal liver metastases. *Aust. NZ. J. Surg.*, **65**, 383–9.

[5] Cooper, I. S. (1993). Cryogenic surgery A new method of destruction or extirpation of benign or malignant tissues. *N. Eng. J. Med.*, **268**, 743–9.

[6] Ravikumar, T. S., Steele, G. Jr., Kane, R. and King, V. (1991). Experimental and clinical observations on hepatic cryotherapy for colorectal metastases. *Cancer Res.*, **51**, 6323–6327.

[7] Seifert, J. and Morris, D. L. (1998). Prognostic factors following cryotherapy for hepatic metastases from colorectal cancer. *Ann. Surgery*, **228**(2), 201–208.

[8] Adam, R., Akpinar, B., Johann, M. *et al.* (1997). Place of cryotherapy in the treatment of malignant liver tumours. *Ann. Surg.*, **225**, 39–50.

[9] Korpan, N. N. (1997). Hepatic cryosurgery for liver metastases. Long-term follow-up. *Ann. Surg.*, **225**, 193–201.

[10] Yeh, K. E., Fortunato, L., Hoffman, J. P. and Eisenberg, B. L. (1997). Cryosurgical ablation of hepatic metastases from colorectal carcinomas. *Am. Surg.*, **63**, 63–68.

[11] Seifert, J. F., Junginger, T. and Morris, D. L., A collective review of the world literature on hepatic cryotherapy. *J. R. Coll. Surg. Edinb.*, June, 1998, **43**, 141–154.

[12] Ross, W. B., Horton, M., Bertolino, P. and Morris, D. L. (1995). Cryotherapy of liver tumours – a practical guide. *HPB Surgery*, **8**, 167–73.

